# Supplementary material for: Hepatocyte-derived IL-10 plays a crucial role in attenuating pathogenicity during the chronic phase of T. congolense infection
Source: PLoS Pathog. 2020 Feb 3;16(2):e1008170. doi: 10.1371/journal.ppat.1008170 (PMC7018099; doi:10.1371/journal.ppat.1008170)
Supplement: S1 Table — (DOCX) [file ppat.1008170.s001.docx]

**Table S1: Fluorescently labeled antibodies used**

| Antibody | Clone | Fluorescent label | Company |
| --- | --- | --- | --- |
| Ter-119 | TER-119 | PE  APC | BD Pharmingen  eBioscience |
| CD71 | R17217 | FITC | eBioscience |
| CD41 | eBioMWReg30 | FITC | eBioscience |
| CD45 | 30-F11 | APC-Cy7 | Biolegend |
| CD11b | M1/70 | PE-Cy7 | Biolegend |
| CD31 | 390 | APC | eBioscience |
| Ly6C | HK1.4 | Pacific Blue | Biolegend |
| Ly6G | 1A8 | Per-CP-Cy5.5 | Biolegend |
| F4/80 | A3-1 | PE | BioRad |
| B220 | RA3-6B2 | AmCyan | Biolegend |
| CD19 | eBio1D3 | PE | eBioscience |
| MHC-II | M5/114.15.2 | Per-CP-Cy5.5  Pacific Blue | BD Pharmingen  Biolegend |
| NK1.1 | PK136 | Per-CP-Cy5.5 | Biolegend |
| CD4 | GK1.5 | Pacific Blue | eBioscience |
| CD8 | 53-6.7 | Amcyan | eBioscience |
