# Supplementary material for: Hepatocyte-derived IL-10 plays a crucial role in attenuating pathogenicity during the chronic phase of T. congolense infection
Source: PLoS Pathog. 2020 Feb 3;16(2):e1008170. doi: 10.1371/journal.ppat.1008170 (PMC7018099; doi:10.1371/journal.ppat.1008170)
Supplement: S2 Table — (DOCX) [file ppat.1008170.s002.docx]

**Table S2: Primer used for RT-PCR analysis**

| Gene name | Sequence |
| --- | --- |
| *IL-10* | F: 5’-ACTCAATACACACTGCAGGTG-3’  R: 5’-GGACTTTAAGGGTTACTTGG-3’ |
| *TNF* | F: 5’-5’-CCTTCACAGAGCAATGACTC-3’  R: 5’-GTCTACTCCCAGGTTCTCTTC-3’ |
| *IL-6* | F: 5’- GTCTTCTGGAGTACCATAGC-3’  R: 5’-GTCAGATACCTGACAACAGG-3’ |
| *Mif* (Macrophage migration inhibitory factor) | F: 5’-CTTTTAGCGGCACGAACGAT-3’  R: 5’-AAGAACAGCGGTGCAGGTAA-3’ |
| *Nos2* | F: 5’-GCTTCTGGTCGATGTCATGAG-3’  R: 5’-TCCACCAGGAGATGTTGAAC-3’ |
| *Arg1* | F: 5’-TTAAAGCCACTGCCGTGTTC-3’  R: 5’-ATGGAAGAGACCTTCAGCTAC-3’ |
| *Cxcl-10* | F: 5’-GAAATTATTCCTGCAAGCCAATTT-3′  R: 5′-TCACCCTTCTTTTTCATGTAGCA-3 |
| *Hamp* | F: 5’-CAGCAGAACAGAAGGCATGA-3’  R: 5’-TGCAACAGATACCACACTGG-3’ |
| *Hmox-1* (Heme oxygenase 1) | F: 5’-GACACCTGAGGTCAAGCACAG-3’  R: 5’-CCACTGCCACTGTTGCCAAC-3’ |
| *Nramp-2* (Slc11a2; solute carrier family 11 (proton-coupled divalent metal ion transporters), member 2; DCT1; DMT1) | F: 5’- TCATGGAGGGATTCCTGAAC-3’  R: 5’-TCCTCCAGCCTATTCCATTG-3’ |
| *Fpn1* (Ferroportin-1) | F: 5’-CCAGTCATTGGCTGTGGTTT-3’  R: 5’-AGGTGGGCTCTTGTTCACAT-3 |
| *Fth1* (Ferritin Heavy Chain) | F: 5’-GTCAGCTTAGCTCTCATCAC-3’  R: 5’-ACGTCTATCTGTCTATGTCTTG-3’ |
| *Lcn-2* | F: 5’- ACTGAATGGGTGGTGAGTGT-3’  R: 5’-TCCAGATGCTCCTTGGTATG-3’ |
| *Tert-1* | F: 5'-ATCTGCAGGATTCAGATGCC-3'  R: 5'-GCAGGAAGTGCAGGAAGAAG-3' |
| *IL-10R* | F: 5'-CTGAGCCTAGAATTCATTGCATACG-3'  R: 5'-TGAGTTTCCGTACTGTTTGAGGG-3 |
