# Supplementary figures and images for: Hepatocyte-derived IL-10 plays a crucial role in attenuating pathogenicity during the chronic phase of T. congolense infection
Source: PLoS Pathog. 2020 Feb 3;16(2):e1008170. doi: 10.1371/journal.ppat.1008170 (PMC7018099; doi:10.1371/journal.ppat.1008170)

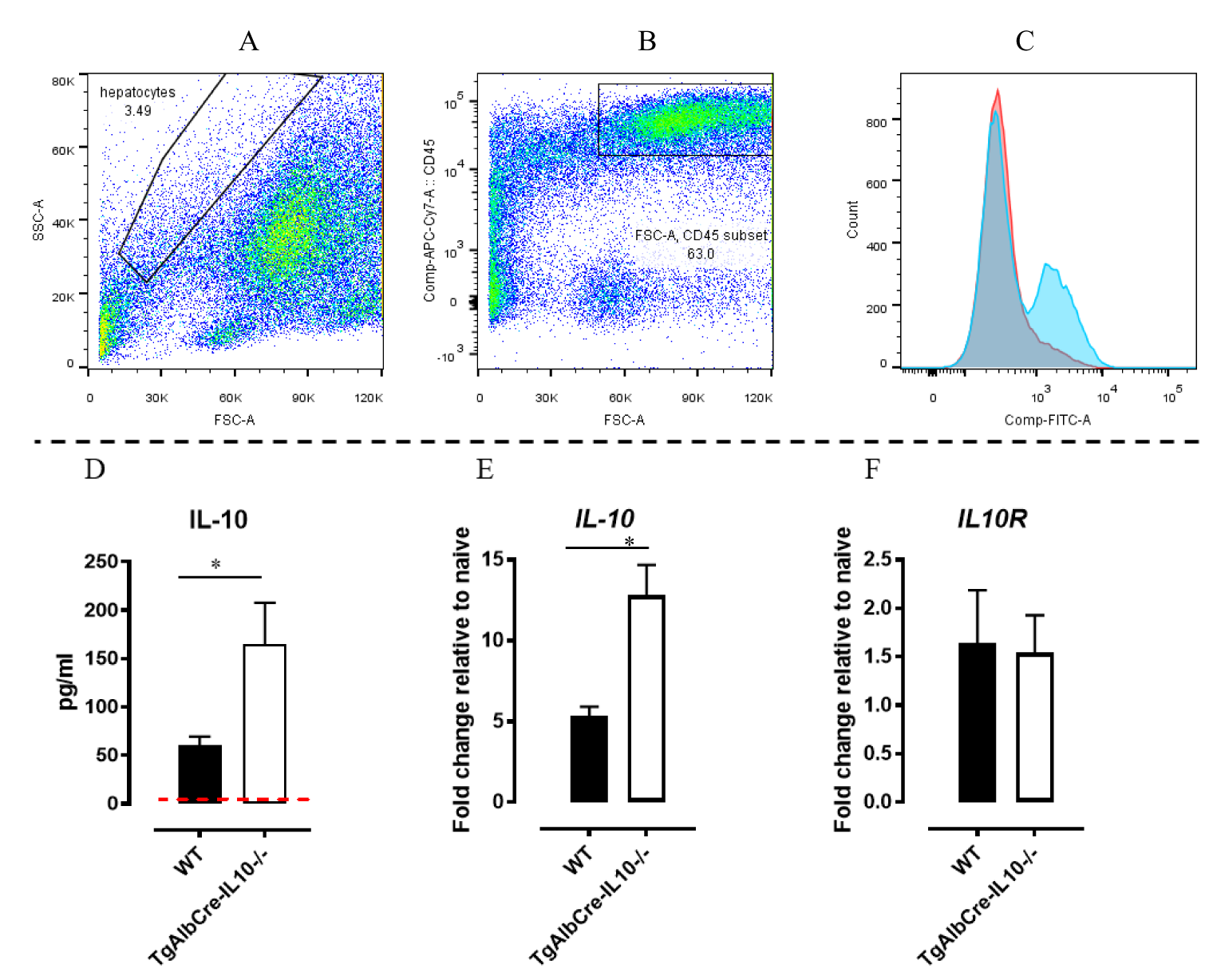

Supplement: S1 Fig — Representative FACS profile of purified leukocytes at day 45 post T. congolense infection. The gating strategy used to discriminate between leukocytes and potential contaminating hepatocytes/debris is based on an FSC-A versus SSC-A (A) and a CD45 versus FSC-A (B) plot, whereby leukocytes were selected based on their CD45+ profile. (C) Histogram plot showing the intensity of the IL-10-eGFP signal in leukocytes from IL-10-eGFP reporter (blue) mice and, as negative control, in leukocytes from TgAlbCre-IL10-/- (red) mice. At day 45 post T. congolense infection, isolated hepatocytes from WT (black symbol) and TgAlbCre-IL10-/- (white symbol) mice were cultured for 36 hours and subsequently tested in ELISA for IL-10 protein levels (D) or tested in RT-PCR for IL-10 and IL-10R gene expression (E and F, respectively). Of note, RT-PCR results are presented as fold change whereby the expression levels were normalized using S12 and expressed relatively to the expression levels in the corresponding non-infected animals. Non-infected animals did not show any detectable IL-10 protein levels (Dashed line). Data are represented as mean of at least 3–5 mice per group ± SEM and are representative of 2 independent experiments. (*: p≤0.05, **: p≤0.01, ***: p≤0.005). ND: Not detected. (TIF) [file ppat.1008170.s003.tif]

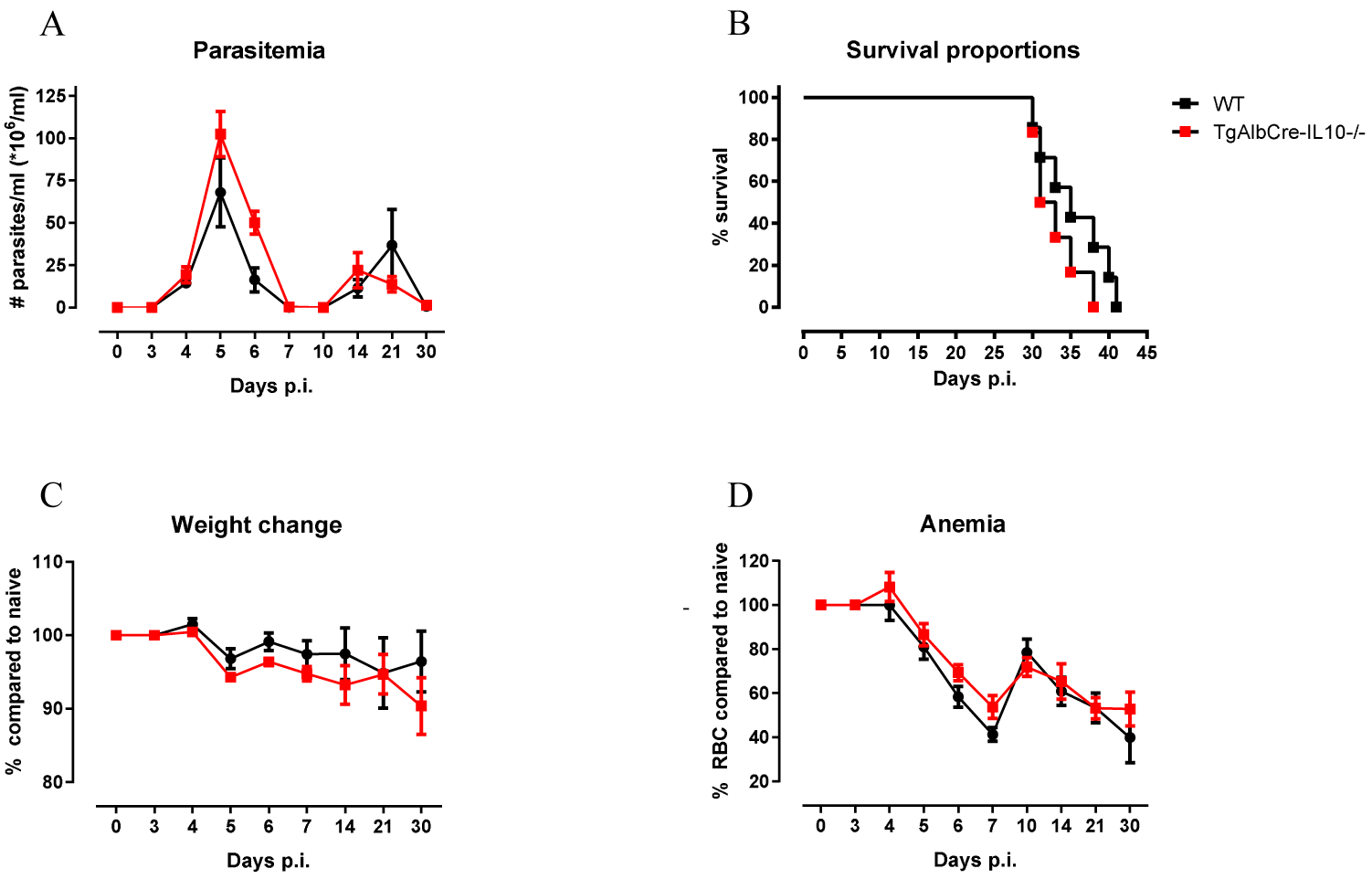

Supplement: S2 Fig — A) Parasitemia, (B) Survival, (C) weight change, (D) anemia of T. brucei infected wild type (WT, black symbol) and TgAlbCre-IL10-/- (red symbol) mice. Data are represented as mean (A, C-G) or median (B) of 3–5 mice per group ± SEM and are representative of 2–3 independent experiments. (TIF) [file ppat.1008170.s004.tif]

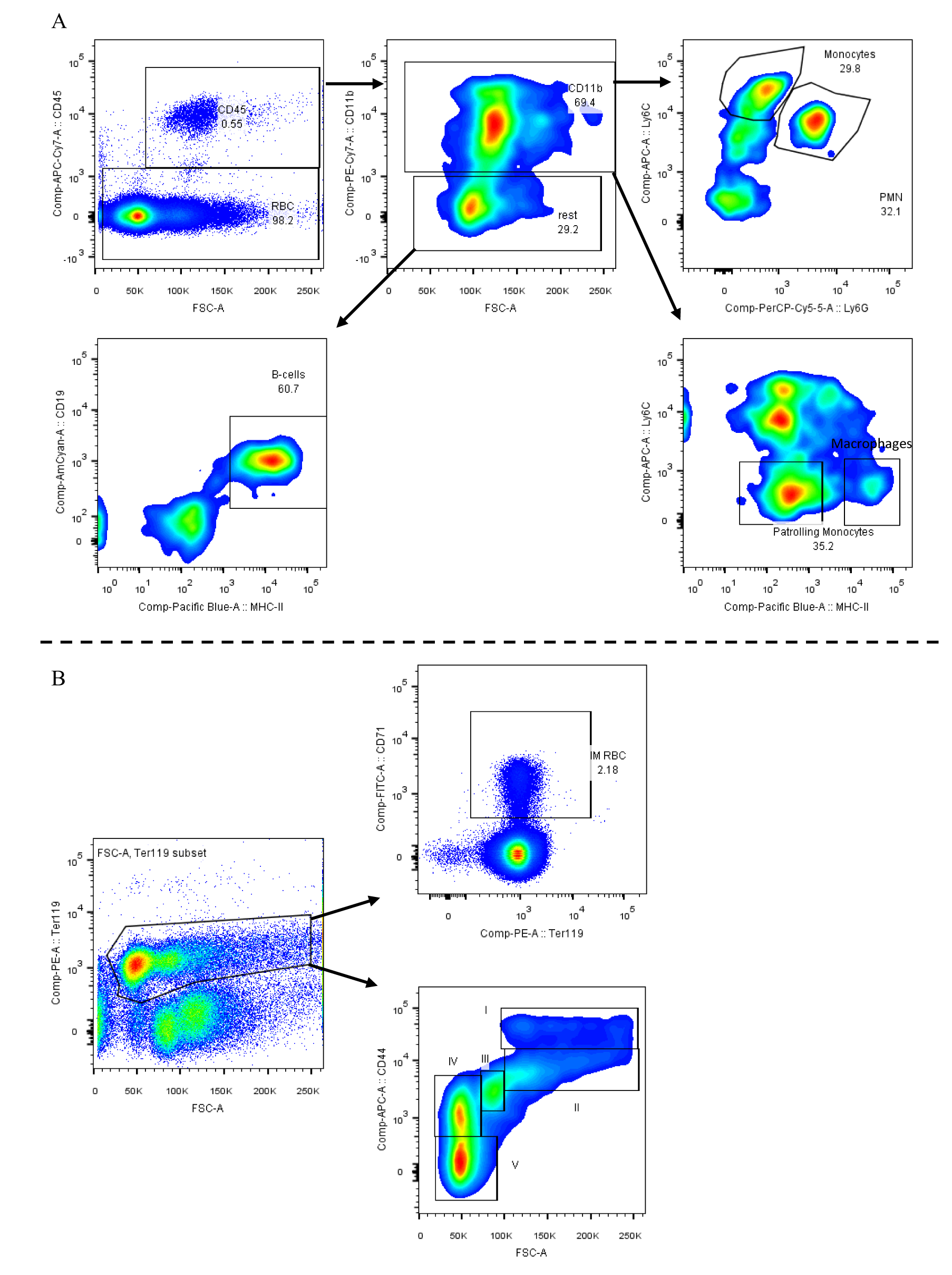

Supplement: S3 Fig — Representative FACS profiles on blood of T. congolense infected animals to identify different leukocyte (A) and RBC (B) subsets. For the leukocyte, first, a CD45 versus FSC-A plot allows identifying CD45+ cells, after which these cells put in a CD11b versus FSC plot to identify CD11b+ cells and CD11b- cells (lymphocytes). The CD11b- cells (lymphocytes) were then plot in a CD19 versus MHC-II plot to identify B cells (CD19+MHC-II+) and T cells (CD19-MHC-II-). The CD11b+ cells were plotted in a Ly6C versus Ly6G plot to identify inflammatory monocytes (Ly6C+Ly6G-) and PMN (Ly6CintLy6G+). Alternatively, the CD11b+ cells were plotted in an Ly6C versus MHC-II plot to identify patrolling monocytes (Ly6C-MHC-II-). Regarding the RBC subsets, a Ter119 versus FSC-A plot allows identification of RBCs (i.e. Ter-119+ cells). These cells be plot in a CD71 versus Ter-119 plot to identify immature (Ter119+CD71+) and mature (Ter119+CD71-) cells, or in a CD44 versus FSC-A plot to identify nucleated erythroblasts (pro- and basophilic (I), polychromatic (II), orthochromatic (III) erythroblasts) from nucleated reticulocytes (IV) and enucleated erythrocytes (V). (TIF) [file ppat.1008170.s005.tif]

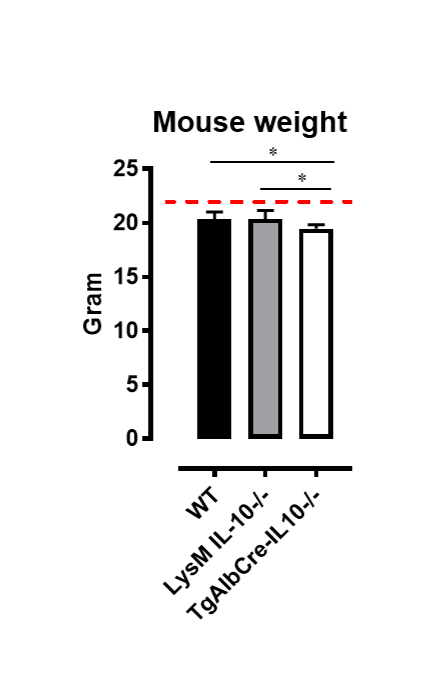

Supplement: S4 Fig — Absolute weights of T. congolense infected wild type (WT, black symbol) and TgAlbCre-IL10-/- (red symbol) mice, when considering (subtracting) the increase in hepatosplenomegaly. Data are represented as mean of 3–5 mice per group ± SEM and are representative of 2–3 independent experiments. (TIF) [file ppat.1008170.s006.tif]

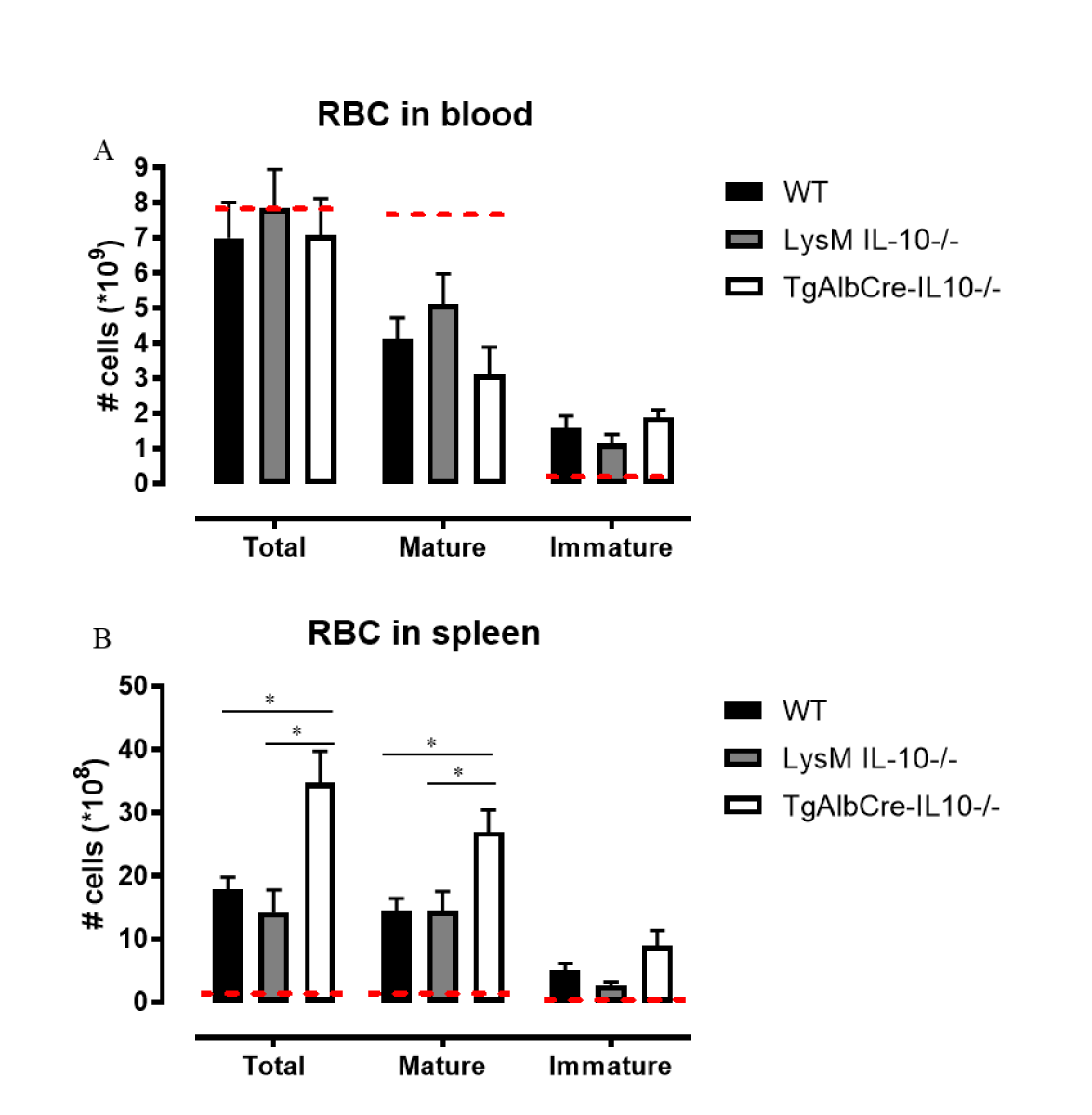

Supplement: S5 Fig — (A) Total number of RBCs as well as mature and immature RBCs in the blood of T. congolense infected (Day 45 p.i.) mice, which were calculated based on the total blood volume (Fig 6A). WT (black symbol), LysM-IL-10-/- (grey symbol) and TgAlbCre-IL10-/- (white symbol) mic. (B) Total number of RBCs as well as mature and immature RBCs in the spleen of T. congolense infected (Day 45 p.i.) mice, Dashed line represents cytokine levels in non-infected animals. Data are represented as mean of at least 3–5 mice per group ± SEM and are representative of 2 independent experiments. (*: p≤0.05, ***: p≤0.005). (TIF) [file ppat.1008170.s007.tif]

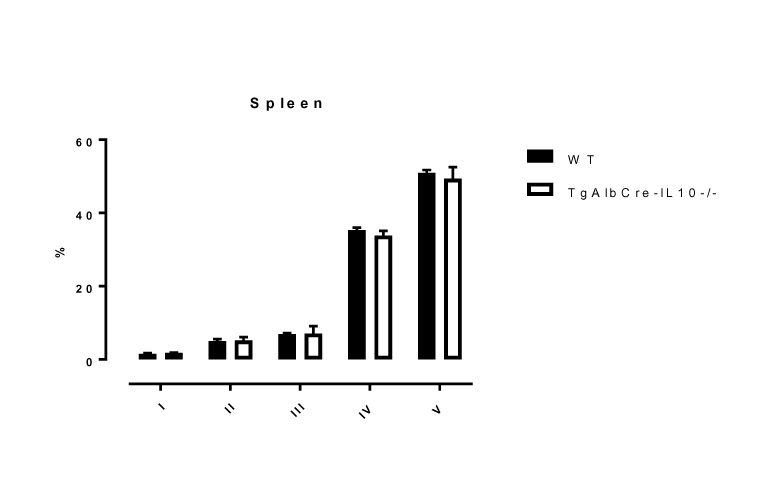

Supplement: S6 Fig — Percentage of the different erythroid populations (defined as described in S3B Fig) in spleen of WT (black bar) and TgAlbCre-IL10-/- (open bar) mice at 40 days p.i. Results are representative of 2 independent experiments and shown as mean of 3 individual mice ± SEM. (TIF) [file ppat.1008170.s008.tif]

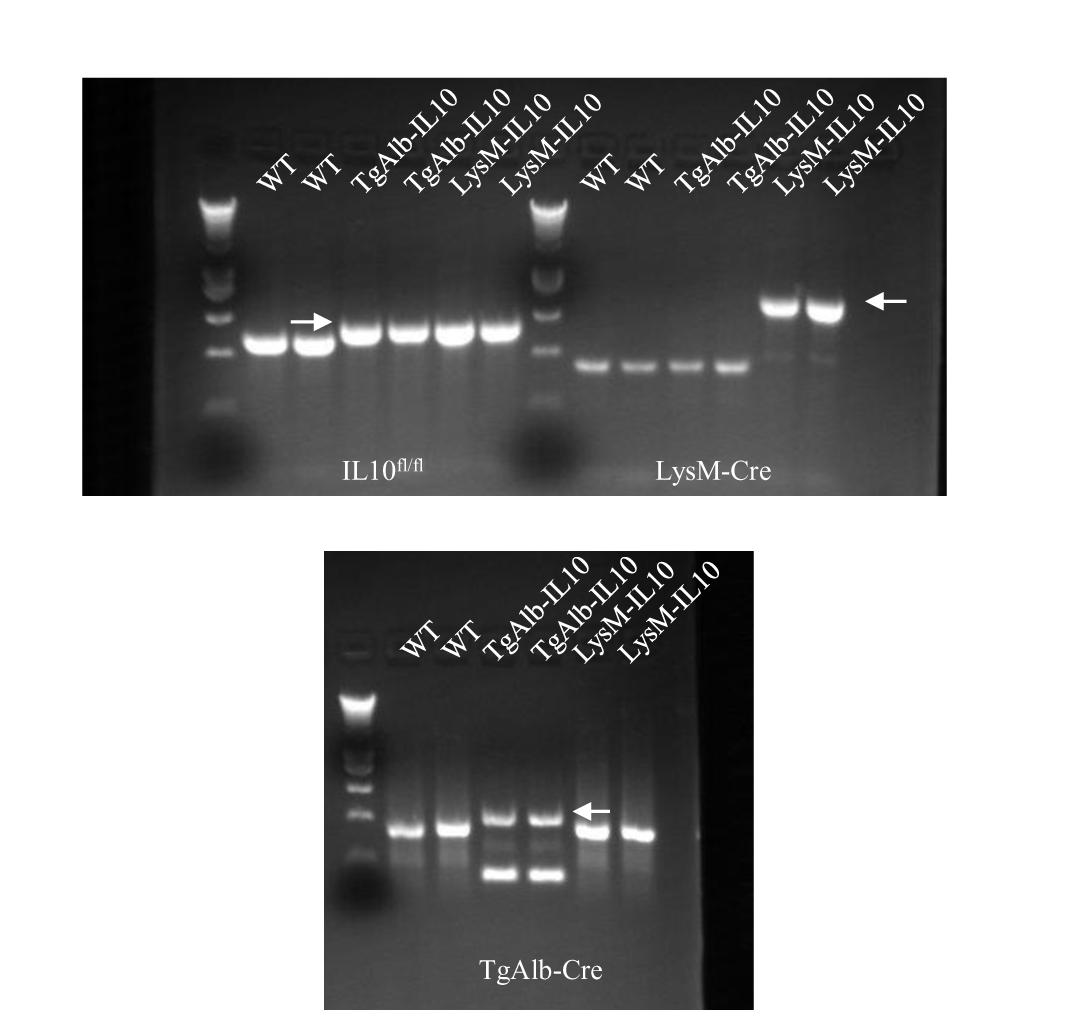

Supplement: S7 Fig — Prior to performing experiments mice were genotyped using the conditions described by the supplier (Jackson mice). Upper left panel: IL10fl/fl genotyping profile, Upper right panel: LysMCre genotyping profile, Lower panel: TgAlbCre genotyping profile. (TIF) [file ppat.1008170.s009.tif]
