# Supplementary material for: Hepatocyte-derived IL-10 plays a crucial role in attenuating pathogenicity during the chronic phase of T. congolense infection
Source: PLoS Pathog. 2020 Feb 3;16(2):e1008170. doi: 10.1371/journal.ppat.1008170 (PMC7018099; doi:10.1371/journal.ppat.1008170)
Supplement: Attachment — Submitted filename: Answers to the reviewers.doc [file ppat.1008170.s010.doc]

**Answers to the reviewers:**

**Major Comments:**

***Reviewer #1:***

*1) The last sentence of the introduction is an overstatement. The data do support that hepatocyte-derived IL-10 is essential, but the authors have not shown that this is the “only” source that is critical. T cell-specific IL-10 KO may very likely result in the same susceptibility phenotype, and the authors have not explored this. This is also relevant to the authors’ lack of clearly defined rationale for experimentally focusing on myeloid-derived IL-10, but not T cell-derived IL-10. That myeloid cells could produce IL-10 is not much of an argument for ignoring T cell-derived IL-10. Have others already used T cell-specific IL-10 deficient genetic systems to show that T cells are (or are not) a biologically relevant source of IL-10 during T. congolense infection? If those experiments have been published, the reference(s) should be provided. More rationale should be provided.*

We agree with the reviewer that other cells besides hepatocytes, *i.e.* T cells and myeloid cells, could be potential sources of IL-10 during the later stage of *T. congolense* infection. So far, there are no publications documenting the contribution of T cells to IL-10 production at these later stages of *T. congolense* infection, but this possibility can indeed not be ruled out. Our own published work showed that myeloid cells could be a potential source of IL-10 during the later infection stage of this parasite, however without affecting the survival of the animals (Bosschaerts et al., IL-10 limits production of pathogenic TNF by M1 myeloid cells through induction of nuclear NF-κB p50 member in Trypanosoma congolense infection-resistant C57BL/6 mice. Eur J Immunol. 2011;41: 3270–3280. doi:10.1002/eji.201041307), Of note, important immunopathological parameters, such as weight loss, anemia and thrombocytopenia, were not assessed in that study and are now included in this manuscript. Hence, we included myeloid-specific IL-10-deficient mice in our current analysis as an internal control, knowing that these cells are producers of IL-10 without majorly impacting survival. In this manuscript, we unequivocally demonstrate that hepatocyte-derived IL-10 does have the capacity to significantly affect survival of the host.

Since we can not formally exclude a role for T-cell-derived IL-10 (or IL-10 from yet other, undefined sources), we omitted the word “only” in the last sentence of the introduction: “Hence, although other cells can produce IL-10 during experimental *T. congolense* infection, hepatocyte-derived IL-10 is crucial to control inflammation-induced immunopathogenicity during the chronic stage of infection that ultimately mediates host survival.”

*2) Figure 4: All of the "significant" cellular and inflammatory perturbations occur after parasite control is lost in TgAlbCre-IL-10-/- mice (after day 18, Figure 1A) and after at least half the TgAlbCre-IL-10-/- mice are dead (day 45, Figure 1B). The authors should attempt to reconcile these data. Why do TgAlbCre-IL-10-/- mice lose control of the infection after the second week? Why are the TgAlbCre-IL-10-/- mice dying? These analyses of cellular and inflammatory perturbations, which are quite superficial, precede neither the loss of parasite control nor the death of the mice so it is not at all surprising that very sick mice with very high parasite burdens exhibit these perturbations.*

So far, the exact cause of death associated with *T. congolense* infection is unknown. However, we believe that a persistent and uncontrolled pro-inflammatory immune response in the infected host will ultimately lead to multiple organ failure. This phenomenon is strongly amplified and accelerated in TgAlbCre-IL-10-/- mice at later stages (after day 38 p.i.) of infection, as it cannot be sufficiently tempered by IL-10 in these mice. Hence, we measured the immunopathogenic parameters of the mice at day 45 of infection exactly to get an insight of their (hyper)inflammatory status at the time of dying. In order to answer the reviewers question (*Why are the TgAlbCre-IL-10-/- mice dying?*) and to draw the attention of the potential link between hyperinflammation and multiple organ failure, we included in the discussion (Page 20) a sentence “In turn, this higher and persistent inflammatory immune response can also contribute to multiple organ failure and host death [19,23].”

We now also attempted to reconcile the question “*Why do TgAlbCre-IL-10-/- mice lose control of the infection after the second week?*” with the early death observed of TgAlbCre-IL-10-/- mice by including following sentences in the discussion (Page 19): “Since antibodies are required to keep the parasitemia in check [11,44], the reduced B-cell presence in the blood could in turn explain the higher parasitemia levels observed in TgAlbCre-IL10^-/-^ mice at later stages of infection.” and “Though the cause of B-cell depletion is currently unknown, it was shown that the kinetics of this B-cell loss and parasite outgrowth depends on different factors such as parasite virulence and the level of inflammation [45,46]. Therefore, the observed reduced B cell numbers in TgAlbCre-IL10^-/-^ mice at the later phase of infection are most likely due to a higher and persistent inflammatory immune response [45,47].”

*Given the critical role of secreted antibody in controlling this parasite, an obvious question relates to whether humoral immunity derailed in the absence of hepatocyte-derived IL-10.* *Measuring total cells in the blood does not reveal whether critical circuits/networks such as germinal center reactions, plasma cells, B cell activation, antibody secretion, CD4+ T follicular helper cell numbers and function, or Th1 cells are impaired.* ***Minimally, some discussion is warranted.*** *Relevant to this point, Page 19 lines 13-14: there are no data to support that reduced B cell numbers could explain the elevated parasitemia, given that B cell numbers remain identical through day 28 to 38 p.i., which is well after the TgAlbCre-IL-10-/- mice have lost control of the parasite (which occurs on 18 p.i., day according to Figure 1A).*

We have published before that B-cells and IgG antibodies are required for parasitemia control (Magez et al., [J Infect Dis.](https://www.ncbi.nlm.nih.gov/pubmed/16652287) 2006 Jun 1;193(11):1575-83. Epub 2006 Apr 21. Interferon-gamma and nitric oxide in combination with antibodies are key protective host immune factors during trypanosoma congolense Tc13 Infections) and that during the course of *T. congolense* infection there is a complete disruption of B-cell homeostasis, strongly suggesting that B-cell networks are affected (Obishakin et al., [Parasite Immunol.](https://www.ncbi.nlm.nih.gov/pubmed/24451010) 2014 May;36(5):187-98. doi: 10.1111/ pim.12099. Chronic Trypanosoma congolense infections in mice cause a sustained disruption of the B-cell homeostasis in the bone marrow and spleen). In order to comply with the reviewer’s request for more discussion, we extended the existing text in the Discussion (Page 19) to: “Furthermore, the number of circulating B cells was found to be similar in both groups of mice and was only significantly reduced in TgAlbCre-IL10^-/-^ mice at later stages of infection. Since antibodies are required to keep the parasitemia in check [11,44], the reduced B-cell presence in the blood could in turn explain the higher parasitemia levels observed in TgAlbCre-IL10^-/-^ mice at later stages of infection. It is however important to realize that B-cell counts in the blood do not reveal whether B-cell activation, the formation of plasma cells and antibody secretion, the induction of germinal center reactions, or the activation of CD4^+^ T follicular helper cell numbers and function, which are known to restrict *T. congolense* parasitemia [11,44], are impaired. However, this is highly likely since trypanosomes cause a B-cell depletion pathology, which is initiated by the very rapid disappearance of immature B cells in the bone marrow, as well as transitional and IgM^+^ marginal zone B cells from the spleen, followed by a gradual depletion of Follicular B cells (FoB) [45,46].”

*3) Figure 12: These data represent a very interesting and exciting attempt to understand the inflammatory, parasitological, morbidity and mortality phenotypes in observed in TgAlbCre-IL-10-/- mice, compared to WT mice. As described, one cohort of TgAlbCre-IL-10-/- mice was treated on day 30 p.i. with regular injections of a nanobody targeting host MIF. The data in Figure 12 show that neutralizing MIF abrogates the exacerbated anemia phenotype observed in T congolense-infected TgAlbCre-IL-10-/- mice but has no effect on modulating parasite burdens. Based on these data the authors argue that MIF signaling mediated the phenotype observed in TgAlbCre-IL-10-/- mice. The authors should specify which phenotype.*

In the text it is clearly stated that “the anti-MIF Nb treatment did not affect parasitemia development in TgAlbCre-IL10^-/-^ mice (Fig 12A, upper panel), but it clearly prevented anemia from further progressing (Fig. 12A, lower panel).”

In order to further clarify our statement and to be more precise, we rephrased the sentence (Page 17) “Collectively, blocking MIF in TgAlbCre-IL10^-/-^ mice, using an anti-MIF Nb, was found to attenuate anemia and excessive systemic inflammatory cytokine levels, indicating that MIF signalling mediates the phenotype observed in TgAlbCre-IL10^-/-^ mice.” into “Collectively, blocking MIF in TgAlbCre-IL10^-/-^ mice, using an anti-MIF Nb, was found to attenuate anemia and excessive systemic inflammatory cytokine levels, without affecting parasite burden.”

*This lack of precision aside, there are concerns regarding the parasitemia data shown in Figure 12A. According to these results, there are no differences in parasite burden in untreated WT and untreated TgAlbCre-IL-10-/- mice on either day 35, 40, or 45 p.i. (days 5, 10, 15 post-treatment, respectively, as shown on the graph). However, Figure 1A shows that by either day 30, 35, 40, or 45 p.i., the TgAlbCre-IL-10-/- mice should exhibit burdens that exceed the WT mice by 80% or more (TgAlbCre-IL-10-/- mice have substantially elevated parasite burdens by days 18, 34, 40 and 48 as shown in Figure 1A). Thus, the data in Figure 12 stand in clear opposition to the data shown in Figure 1A and undermine the robustness of the model and data sets.*

We agree that the kinetics of the parasitemia experiment, comparing WT and TgAlbCre-IL10^-/-^ mice, shown in Figure 12 is somewhat altered as compared to Figure 2A, but the main conclusion remains unaltered: parasite control is eventually lost in TgAlbCre-IL10^-/-^ mice. Figure 2 is representative for at least 3 independent experiments, but we acknowledge that some variations in the kinetics and the extent of parasitemia increase at later stages of infection in TgAlbCre-IL10^-/-^ mice may occur. This was also shown by Obishakin et al. ([Parasite Immunol.](https://www.ncbi.nlm.nih.gov/pubmed/24451010) 2014 May;36(5):187-98. doi: 10.1111/ pim.12099. Chronic *Trypanosoma congolense* infections in mice cause a sustained disruption of the B-cell homeostasis in the bone marrow and spleen). Therefore, we included a sentence in the discussion (Page 19-20): “Though the cause of B-cell depletion is currently unknown, it was shown that the kinetics of this B-cell loss and parasite outgrowth depends on different factors such as parasite virulence and the level of inflammation [45,46]. ”However, since the main conclusions of all experiments are consistent, we feel confident to compare treatment regimens (e.g. anti-MIF) within one given experiment with a given kinetics.

# *The studies in Figure 12 both lack comprehensive experimental control groups (e.g. anti-MIF-treated WT mice and irrelevant/non-specific Nb reagents).*

# We did not perform an anti-MIF Nb treatment in WT mice since the main goal of the experiment was to attenuate the excessive inflammatory immune response observed in TgAlbCre-IL10^-/-^ mice and to evaluate if we could bring this to levels observed in WT mice. This appears to be the case for some, but not all, immunopathological parameters.

# In our previously published work, using the same anti-MIF Nb in comparison to an irrelevant control Nb in a strongly inflammatory endotoxic shock model, the anti-MIF Nb was found to be very effective in blocking inflammation while an irrelevant control Nb did not show any effect. Also no unwanted bystander effects were observed when using this control Nb (Sparkes et al., Novel half-life extended anti-**MIF** nanobodies protect against endotoxic shock, [**FASEB J.**](https://www.ncbi.nlm.nih.gov/pubmed/29401625) 2018 Jun;32(6):3411-3422. doi: 10.1096/fj.201701189R. Epub 2018 Jan 25). Therefore, we do not anticipate this control Nb to have any effect in the current research.

***Reviewer #2:***

No further experiments required

**Minor comments:**

***Reviewer #1:***

*1) Typographical error, Page 7 line 5. Extend should be “extent.”*

This has been corrected in the revised version.

*2) Page 8 line 5: Either data should be shown, or a reference should be provided to support the statement that, “levels of IL-10 progressively increase during the course of infection…”*

We included in the revised version of our article a reference documenting on the fact that IL-10 levels progressively increase during the course of infection (Guilliams et al., [J Immunol.](https://www.ncbi.nlm.nih.gov/pubmed/17709488) 2007 Sep 1;179(5):2748-57) and the sentence reeds “ levels of IL-10 progressively increase during the course of infection in order to sustain host survival [19]. Accordingly, the reference list has been updated.

*3) The authors argue that IL-10 functions in an autocrine manner to potentiate IL-10 production in hepatocytes. This should be shown by either culturing hepatocytes with recombinant IL-10 or blocking IL-10R signaling in the cultures treated with either lysate, parasites, or VSG.*

We agree with the reviewer that potentially autocrine effects of IL-10 could be tested by using recombinant IL-10 or adding blocking IL-10R antibodies to hepatocyte cultures.

Since the editors proposed to address the questions without performing additional experiments, we prefer to omit this statement from the manuscript (page 8, last sentence of the last paragraph). In addition, we softened a sentence in the discussion ‘page 21) into “Moreover, hepatocytes from WT infected mice, but not TgAlbCre-IL10^-/-^ infected mice, exhibited an increased *IL-10R* gene expression, suggesting that autocrine/paracrine IL-10R signalling on hepatocytes may be required to allow efficient control over inflammation. Additional experimentation will be required to prove this point.”

*4) The legend for Figure S1 states that TgAlbCre-IL-10-/- mice did not show any detectable IL-10 protein levels. However, the graph shows substantial proteins levels from leukocyte pools from the TgAlbCre-IL-10-/- mice. Please clarify*.

This was a mistake and is corrected as ‘Non-infected animals did not show any detectable IL-10 protein levels (Dashed line).”

*5) In Figure 3, the dashed red line should be defined. Do these lines denote the limit of detection of the assay, or levels measures in naïve mouse sera?*

We clarified this point in the legend of Figure 3 (Page 31) of the revised version: “Dashed line represents cytokine levels in non-infected animals”.

*6) Why are the mice infected with T. brucei dying? It appears to have nothing to do with parasitemia (Fig. S2A), which therefore would leave inflammatory immunopathology as the likely cause, which similar to the T. congolense model, would be expectedly counteracted by IL-10. Why might hepatocyte-derived IL-10 not matter after day 25-20 in the T. brucei experimental system?*

Though we do not know the exact cause of death associated with *T. brucei* infection, we believe that a persistent and uncontrolled pro-inflammatory immune response will lead to multiple organ failure and hence culminate in host death. There are however several major differences between *T. brucei* and *T. congolense* infections. Indeed *T. brucei* is an extravascular parasite, not only causing anemia and liver injury, but also neuroinflammation as important pathological feature (Sternberg et al., [J Neuroimmunol.](https://www.ncbi.nlm.nih.gov/pubmed/16054238) 2005 Oct;167(1-2):81-9). This is associated with a persistently high pro-inflammatory immune response throughout the infection, during which only very low levels of IL-10 are recorded (Stijlemans et al., [PLoS Pathog.](https://www.ncbi.nlm.nih.gov/pubmed/25255103) 2014 Sep 25;10(9):e1004414. doi: 10.1371/journal.ppat.1004414. eCollection 2014 Sep.). Conversely, *T. congolense* is a strictly intravascular parasite with anemia and liver injury as most prominent features, which is characterized by an early pro-inflammatory immune response that switches to an IL-10-dependent anti-inflammatory immune response as the disease progresses to a more chronic phase.

*7) Figure 5D: It is not clear that the IFN-g, TNF, MIF and IL-6 serum data collected on day 45 p.i. represent fundamentally new information given that the relative cytokine levels and relationships were established on day 48 as part of Figure 3.*

It is correct that cytokine levels at day 45 p.i. are represented in Figure 3 (in which a full kinetic of cytokine production is shown) as well as in Figure 5D. Importantly, Figure 5D shows an independently generated data set, which is an integral part of the message we want to bring in Figure 5. To keep coherence in this figure, we prefer to maintain Figure 5D as it is.

***Reviewer #2:***

*The authors showed that also IL-6 is very differently regulated (indeed more than MIF). Some information should be given why they concentrate on MIF and not IL-6. Indeed, new therapeutic strategies to modify IL-6 in humans are already near to clinical application.*

Indeed, the gene and protein expression levels of IL-6 were found to be higher than those of MIF (Fig. 9a and 10a, respectively) in hepatocytes from hepatocyte-specific IL-10 deficient mice. Hence, we agree with the reviewer that IL-6 would also be a relevant cytokine to study.

However, we focused on MIF for several reasons:

(i) Our earlier published work showed that MIF is a key player during *T. congolense*-associated pathogenicity (Stijlemans et al., PLoS Pathog. 2016 Sep 15;12(9):e1005862. doi: 10.1371/journal.ppat.1005862. eCollection 2016 Sep.). Indeed, absence of the pro-inflammatory regulator MIF, using *Mif*-deficient mice, was shown to attenuate the inflammatory immune response during the chronic stage of infection resulting in a reduced infection-associated pathogenicity (*i.e.* anemia, liver and tissue injury) and prolonged survival by promoting IL-10 production.
(ii) Since the data using MIF-/- deficient mice are opposite compared to the data obtained using hepatocyte-specific IL-10 deficient mice, and since we have unique in house blocking anti-MIF Nbs available, we focused on MIF as prime candidate.
(iii) Moreover, in contrast to IL-6, MIF is an upstream regulator of the inflammatory cascade whose blockade may be more efficient at attenuating the inflammatory immune response. Hence, a sentence is included on page 16 (results section) and in the discussion page 20.
